# Energy availability discriminates clinical menstrual status in exercising women

**DOI:** 10.1186/s12970-015-0072-0

**Published:** 2015-02-19

**Authors:** Jennifer L Reed, Mary Jane De Souza, Rebecca J Mallinson, Jennifer L Scheid, Nancy I Williams

**Affiliations:** Department of Kinesiology, Women’s Health and Exercise Laboratory, 108 Noll Laboratory, The Pennsylvania State University, University Park, PA 16802 USA; Current address: Division of Prevention and Rehabilitation, University of Ottawa Heart Institute, 40 Ruskin Street, Ottawa, ON K1Y 4W7 Canada; Current address: Department of Pediatrics, School of Medicine and Biomedical Sciences, University at Buffalo, Buffalo, NY USA

**Keywords:** Energy balance, Females, Exercise training, Dietary energy intake, Resting metabolic rate, Total triiodothyronine

## Abstract

**Background:**

Conditions of low energy availability (EA) (<30 kcal/kgLBM) have been associated with suppressed metabolic hormones and reductions in LH pulsatility in previously sedentary women during short-term manipulations of energy intake (EI) and exercise energy expenditure (EEE) in a controlled laboratory setting. The purpose of this study was to examine if EA, defined as EA = (EI-EEE)/kgLBM, is associated with disruptions in ovarian function in exercising women.

**Methods:**

Menstrual status was confirmed with daily measures of urinary reproductive metabolites across 1–3 menstrual cycles or 28-day monitoring periods. EA was calculated for exercise days using EI from 3-day diet logs, EEE from heart-rate monitors and/or exercise logs for a 7-day period, and body composition from DXA. Resting energy expenditure (REE) was measured by indirect calorimetry. Total triiodothyronine (TT_3_) was measured from a fasting blood sample.

**Results:**

91 exercising women (23.1 ± 0.5 years) were categorized clinically as either exercising amenorrheic (ExAmen, n = 30), exercising oligomenorrheic (ExOligo, n = 20) or exercising eumenorrheic (ExEumen, n = 41). The eumenorrheic group was further divided into more specific subclinical groups as either exercising ovulatory (ExOv, n = 20), exercising inconsistent (ExIncon, n = 13), or exercising anovulatory (ExAnov, n = 8). An EA threshold of 30 kcal/kgLBM did not distinguish subclinical menstrual status (*χ*^2^ = 0.557, p = 0.46) nor did EA differ across subclinical disturbance groups (p > 0.05). EA was lower in the ExAmen vs. ExEumen (30.9 ± 2.4 vs. 36.9 ± 1.7 kcal/kgLBM, p = 0.04). The ratio of REE/predicted REE was lower in the ExAmen vs. ExEumen (0.85 ± 0.02 vs. 0.92 ± 0.01, p = 0.001) as was TT_3_ (79.6 ± 4.1 vs. 95.3 ± 2.9 ng/mL, p = 0.002).

**Conclusions:**

EA did not differ among subclinical forms of menstrual disturbances in a large sample of exercising women, but EA did discriminate clinical menstrual status, i.e., amenorrhea from eumenorrhea.

## Introduction

A recent study on the prevalence of menstrual disturbances in exercising women reported that 52% of exercising women experience subtle menstrual disturbances such as luteal phase defects and anovulation, and 37% of exercising women may be amenorrheic [1,2]. In women with exercise associated menstrual disturbances (EAMD), a spectrum of clinical sequelae has been observed and includes low bone mineral density [3], stress fractures [4], disordered eating [5], altered vascular function [6], reductions in resting energy expenditure (REE) [7], and suppressed metabolic hormone concentrations [7]. Prospective exercise training studies show that low energy availability (EA) is causally related to menstrual disturbances in exercising females [8-12], as increases in EA are associated with the reversal of menstrual disturbances when exercise training persists [13].

EA has been operationally defined as dietary energy intake (EI) minus exercise energy expenditure (EEE) normalized to kilograms of lean body mass (LBM) i.e., (EA = (EI-EEE)/kg LBM) [14]. This variable represents the net input of energy to the body remaining after exercise training for all other metabolic processes such as reproduction, thermoregulation, cellular maintenance, locomotion, and growth [15]. Conditions of low EA (<30 kcal/kg LBM) have been associated with reduced concentrations of metabolic hormones [14], unfavorable alterations in bone markers [16], and reductions in luteinizing hormone (LH) pulsatility in previously sedentary women during short-term manipulations of EI and EEE in a controlled laboratory setting [14]. Although these studies [14,16] define a critical EA threshold of 30 kcal/kg LBM for the initiation of reduced LH pulsatility, few investigators have examined whether an EA of 30 kcal/kg LBM discriminates normal ovarian function from either subtle (luteal phase defects or anovulation) or severe (oligomenorrhea or amenorrhea) menstrual disturbances in exercising women. Severe reductions in LH pulsatility have been observed in amenorrheic runners [17]. Reductions in LH pulsatility have also been documented in exercising women with eumenorrhea [18], anovulatory menstrual cycles [19], and oligomenorrhea [17]. It is unclear if an EA of 30 kcal/kg LBM or lower is associated with a dose response relationship with menstrual disturbances of increasing severity progressing from luteal phase defects and anovulatory menstrual cycles to oligomenorrhea, and amenorrhea. It seems reasonable to expect that more severe reductions in EA would result in a greater degree of reproductive suppression to prevent the investment of energy directed towards pregnancy and away from life sustaining processes. Along these lines, we have previously published that the degree of suppression of REE was a significant predictor of the degree of severity of menstrual disturbances associated with exercise [7].

To produce a defined level of EA, Loucks et al.[14] controlled EI with a clinical dietary product (Ensure Plus, Ross Laboratories, Columbus, OH) and used indirect calorimetry to monitor the target level of EEE. These methods to estimate EI and EEE are not feasible or practical for most free living exercising women. In the few studies [2,4,20-24] that have examined EA in free living exercising women, EA has been defined inconsistently (e.g. kcal/kg LBM, kcal/kg FFM, kcal/kg body weight) and measured using a variety of methods. Accelerometers [2,20,22], indirect calorimeters [20], heart rate monitors [21,23], and physical activity logs [4,21,23,24], most of which analyzed with the Ainsworth compendium [4,21,23], have been used to estimate EEE. Likewise, a number of methods such as dual energy x-ray absorptiometry (DXA) [4,20,21,23], air-displacement plethysmography [22], bioelectrical impedance [24], and skin fold measurements [2] have been used to estimate body composition. Most studies have used self-reported diet logs for various lengths of time to estimate EI [2,21,24]. Due to the small sample sizes [20,22] and inconsistencies in the methods used in the above mentioned studies [2,4,20-24], the role of low EA as a predictor of menstrual abnormalities remains unknown. Conventional methods available to most exercising women such as diet logs, exercise logs, and or heart rate monitors to estimate EA, defined as EI minus EEE relative to LBM [14], should be used to test the role of EA in predicting menstrual status in a large sample of free living exercising women.

The purpose of the current study was to examine whether EA when assessed with conventional methodologies i.e. self-reported diet logs, exercise logs, and heart rate monitoring discriminates both clinical and subclinical menstrual disturbances in ovarian function in premenopausal exercising women. We hypothesized the following: 1) when participants are grouped according to their clinical menstrual status, EA would distinguish the amenorrheic and eumenorrheic exercising women; 2) when participants’ menstrual status is characterized in more detail using hormonal measures, a significantly higher proportion of exercising women will have menstrual disturbances (amenorrhea, oligomenorrhea, anovulation, or luteal phase defects) when their EA is below 30 kcal/kg LBM when compared to the proportion of exercising women who will have menstrual disturbances when their EA is at or above 30 kcal/kg LBM; and 3) below an EA of 30 kcal/kg LBM, menstrual cycle disturbances will progress in severity from luteal phase defects to anovulatory menstrual cycles to oligomenorrhea, and amenorrhea as EA becomes incrementally lower. A secondary purpose was to compare EA values with laboratory measures of metabolic status such as REE and circulating TT_3_ concentrations to determine whether EA measures discriminated menstrual status similarly when compared to more objective laboratory measures of adaptation to energy deficiency.

## Methods

### Experimental design

To assemble the largest data set possible, we combined data from two studies, one performed at the University of Toronto and one performed at The Pennsylvania State University. Both studies were designed to examine menstrual disturbances and alterations in energy balance in exercising premenopausal women and used very similar methodological approaches. The current study includes data from 91 exercising women in whom assessments of menstrual status and EA were performed. Measurements of the variables of interest, i.e., menstrual status, body composition, EI, EEE, and demographics were conducted by the same investigators at both sites using similar methods. The usefulness of EA (assessed with conventional methods) to discriminate menstrual status was compared to laboratory measures of energy status (fasting TT_3_, REE, and ratio of actual to predicted REE [REE/pREE]) [5].

### Participants

Ninety-one participants were recruited by fliers posted on campus and in the surrounding community, newspaper advertisements, and classroom announcements targeting exercising women for a study on women’s health. Initial eligibility criteria included: 1) no history of or current serious medical conditions; 2) no current clinical diagnosis of an eating or psychiatric disorder based on self-report or an interview with a clinical psychologist or licensed clinical social worker; 3) age 18–35 years; 4) non-smoking; 5) no medication use including contraceptives that would alter metabolic or reproductive hormone concentrations; 6) ≥2 hrs/wk of exercise training; and, 7) no history of or clinical diagnosis of polycystic ovarian syndrome (PCOS) and/or not having a free androgen index (FAI), calculated as (total testosterone (nmol/L)/sex hormone binding globulin (SHBG)(nmol/L))*100), > 6 in participants in which these hormones were measured. An FAI greater than 6 has been reported to be consistent with hyperandrogenemia [25] and represents values greater than three standard deviations in our reference sample (n = 37) which consisted of healthy premenopausal exercising women (18–35 years) with documented ovulatory menstrual status [1].

### Screening procedures

Participants were informed of the purpose, procedures, and potential risks of participation in the study before signing an informed consent approved by either the Human Ethics Board at the University of Toronto or the Institutional Review Board at the Pennsylvania State University. Height and weight were measured, and participants completed questionnaires to assess demographics, medical history, exercise history, menstrual history, eating behaviors, bone health, and mental health. A physical exam was performed on all participants by an on-site clinician to determine overall health and check for physical symptoms of PCOS such as acne or hirsutism. In most participants a fasting blood draw was analyzed for a complete blood count (n = 57), basic chemistry panel (n = 35), and an endocrine panel (n = 59). The latter included measures of LH, follicle stimulating hormone, thyroid stimulating hormone, thyroxine, prolactin, dihydroepiandrosterone (Quest Diagnostics, Pittsburgh, PA), total testosterone, and SHBG to rule out illness or endocrine or metabolic disease for most participants. Participants met with a General Clinical Research Center (GCRC) registered dietitian or trained laboratory personnel to receive instructions for completion of 3-day diet logs (2 weekdays and 1 weekend day). Additionally, DXA scans were performed to assess body composition.

### Aerobic capacity

Peak aerobic capacity (VO_2peak_) was measured on a treadmill by indirect calorimetry using an on-line MedGraphics Modular VO_2_ System (St Paul, MN) or SensorMedics Vmax metabolic cart (Yorba Linda, Calif., USA) during the study period using methods that have previously been published [2,7].

### Resting energy expenditure

REE was measured on a single occasion during the study period for all participants. REE was determined by indirect calorimetry using a ventilated hood system (SensorMedics Vmax Series, Yorba Linda, CA, USA). Participants arrived between 0600 and 0900 in a 12 hour post absorptive state within 90 minutes after awakening, having not exercised or ingested caffeine within 24 hours and having not consumed food within 12 hours. In our laboratory, the coefficient of variation for REE measurements, established in 81 participants aged 18–35 years who underwent 2 repeated measurements about 2 weeks apart, was 5.3% ± 0.7%. We compared laboratory assessed REE with a predicted REE (pREE) using the Harris-Benedict equation to estimate how much each individual’s measured REE deviated from the pREE [5]. A lower measured to predicted REE ratio has been associated with a high drive for thinness and with menstrual disturbances in exercising women [5].

### Anthropometrics

Total body weight was measured by a digital scale each week for 4 weeks in the laboratory to the nearest 0.01 kg wearing t-shirt and gym shorts. The mean of these measurements is presented. BMI was calculated as a ratio of weight to height (kg/m^2^). Height was measured to the nearest 1.0 cm without shoes. Body composition, including percent body fat, fat mass (FM), fat free mass (FFM), and LBM was analyzed by a certified technician using DXA on one of three machines. The majority of participants were scanned on either a GE Lunar Prodigy DXA scanner (n = 57) (GE Lunar Corporation, Madison, WI, enCORE 2002 software, version 6.50.069) or a GE Lunar iDXA scanner (n = 26) (General Electric Lunar Corporation, Madison, WI, enCORE 2008 software version 12.10.113). Remaining participants were scanned on a Hologic QDR4500 DXA scanner (n = 8) (Hologic Inc., Bedford, MA). Consistent with the International Society of Clinical Densitometry guidelines, cross calibration studies were performed to remove systematic bias between the systems. For the cross calibration study between the Lunar Prodigy and Lunar iDXA, fourteen participants were scanned in triplicate on both machines. The majority (n = 8) were scanned on both machines within 5 days while approximately one month lapsed between scans for some participants (n = 6). The values for body composition obtained on each scanner were found to be highly correlated (r = 0.97 BF%, r = 0.99 FM, and r = 0.93 LBM) with no significant difference between the sample mean values. For the cross calibration study between the Hologic QDR4500W and the Lunar iDXA, 32 participants were scanned in duplicate on both machines on the same day. Equations were derived using simple linear regression to remove biases, and body composition values obtained from both the Lunar Prodigy and the Hologic QDR-4500 W were calibrated to the Lunar iDXA.

### Menstrual status

The classification of menstrual status was based on self-reported menstrual histories, menstrual calendars used to chart menstrual symptoms i.e., cramps, bleeding, spotting, discharge, etc., and daily measurements of urinary estrone-1-glucoronide (E1G), pregnanediol glucuronide (PdG), and mid-cycle LH profiles. Participants who self-reported eumenorrheic menstrual status, defined as regular menstrual cycle intervals of 26–35 days [1], were monitored for 1-3 menstrual cycles. Participants who self-reported no menses within the past 3 months or 6 or fewer menses within the past year collected first morning urine samples beginning on an arbitrary day for 28 days.

Ovulatory status was confirmed by the presence of a urinary LH peak, identified as a peak concentration above 25 mIU/ml occurring after a mid-cycle E1G peak greater than 35 ng/ml, and followed by a peak luteal phase PdG concentration above 5 μg/ml in participants who exhibited menstrual cycles of 26–35 days [1]. Luteal phase defects were confirmed when the luteal phase was either less than 10 days (short) or when the sum of the 3 day mid luteal peak PdG (sum of mid luteal peak PdG ± 1 day) was less than 10 μg/ml and when the PdG peak concentration was below 5 μg/ml but greater than 2.5 μg/ml in participants who exhibited menstrual cycles of 26–35 days (inadequate) [1]. Anovulatory cycles were confirmed as cycles in which a minimal increase in EIG was observed concomitantly with a failure of LH to rise at midcycle and when a luteal phase exhibited no increase in PdG concentration above 2.5 μg/ml in participants who exhibited menstrual cycles of 26–35 days [1]. Oligomenorrhea was confirmed if menses occurred at intervals of 36–90 days and if participants self-reported 6 or less menstrual cycles in the last year prior to the study. Lastly, functional hypothalamic amenorrhea was assessed by confirming a negative pregnancy test, no menses in the past 90 days, and chronically suppressed E1G and PdG profiles [1].

After consideration of menstrual status, participants were grouped as follows: exercising eumenorrheic (regular menstrual cycle intervals of 26–35 days) (ExEumen, n = 41), exercising oligomenorrheic (inconsistent and long menstrual cycle intervals of 36–90 days) (ExOligo, n = 20), and exercising amenorrheic (no menses for a minimum of 90 days prior to the study and for the duration of the study period) (ExAmen, n = 30), i.e., based on “clinical” menstrual status. The eumenorrheic group was further divided according to detailed characterizations of ovulatory status i.e., based on “subclinical” menstrual status as follows: a) exercising ovulatory (ExOv, n = 20): consistently ovulatory cycles for the duration of the 1–3 menstrual cycle collection period, b) exercising with inconsistent presentations of subtle menstrual disturbances (ExIncon, n = 13): various inconsistent combinations of ovulatory, luteal phase defects, and anovulatory cycles from cycle to cycle for the duration of the 1–3 menstrual cycle collection period, and c) exercising anovulatory (ExAnov, n = 8): consistently anovulatory menstrual cycles for the duration of the 1–3 menstrual cycle collection period.

### Dietary energy intake

Measures of EI were calculated from 3-day diet logs completed during the study period. Participants were provided with a food scale (ECKO Kitchen Scale) and/or food amounts packet. The packet contained diagrams illustrating container sizes, cuts of meat, and various circles and squares which are typically used when estimating portion size for foods. Participants were encouraged to use these scaled diagrams as a guide for describing dimensions and sizes. Also, included in the packet was a sample page of an accurately completed diet record provided as a reference. Participants were asked to record all foods and beverages consumed on 2 weekdays and 1 weekend day. EI was assessed for 3 days instead of 7 as a shorter 3-day diet log has been shown to reduce participant burden and improve compliance [26]. All 3 days of the diet logs were used. Registered dietitians and/or trained laboratory personnel instructed each participant on how to record EI and then later reviewed diet logs with participants for completeness and accuracy. The nutrient data from the 3-day diet logs were coded and analyzed using Nutritionist Pro (Version 3.1, Axxya Systems, Stafford, TX) or the Nutrition Data System for Research (NDSR 2008 Version; University of Minnesota; Minneapolis, MN).

### Exercise energy expenditure

Participants completed exercise logs where all purposeful exercise sessions greater than 10 minutes in duration with a heart rate above 90 beats per minute were recorded for a 7 day period. The 7-day exercise logs were completed the same week as the 3-day diet logs, and all 7 days of the exercise logs were used. Purposeful exercise included activities such as elliptical, running, or strength training, but not daily living activities such as house cleaning or walking a dog. Participants were asked to wear a Polar S610 or RS400 heart rate monitor during all of their purposeful exercise sessions during the 7 day period. Energy expended during these purposeful exercise sessions was measured using the OwnCal feature of the Polar S610 or RS400 heart rate monitors (Polar Electro Oy, Kempele, Finland) [27]. The OwnCal feature has been validated for calculating EEE from heart rate in University age females running, cycling, and rowing at various submaximal intensities [27]. This feature uses body weight, height, age, gender, VO_2peak_, individual maximum heart rate (obtained from VO_2peak_ test or calculated using 220-age), individual heart rate in a sitting position, and heart rate during exercise to derive kilocalories from energy expenditure. Actual VO_2peak_ values were input into the heart rate monitors to compute EEE. The Polar S601 and RS400 hear rate monitors include rest in their estimation of energy expenditure. To estimate only EEE, we subtracted measured REE (kilocalories/min) from the Polar heart rate monitors estimation of energy expenditure. For the few (<20%) purposeful exercise sessions in which participants did not wear the heart rate monitors, the Ainsworth et al. [28] compendiums of physical activities were used to determine the appropriate metabolic equivalent (MET) level for the exercise performed. To calculate the energy expended during the exercise session, the MET level was multiplied by the duration (min) of the exercise session. The MET value includes a resting component. To estimate only EEE, we therefore subtracted measured REE (kilocalories/min) from this value. Determination of MET levels from exercise logs from both experimental sites was made by the same member of the research team.

### Energy availability

EA was operationally defined as EI minus EEE normalized to kilograms of LBM (EA = (EI–EEE)/LBM) [14] and calculated using the data described above for EI, EEE, and LBM. We calculated EA using the averages of each participant’s values for EI, EEE on workout days only, and the LBM from the DXA scan. EEE represents only those calories attributable to exercise in that the estimate of the calories expended for REE throughout the duration of purposeful exercise sessions was subtracted from the estimate of EEE using the Polar heart rate monitors and or physical activity logs.

### Urinary hormone measurements

All urine samples were corrected for specific gravity using a hand refractometer (NSG Precision Cells) to account for hydration status [29] which has been reported to perform as well as creatinine correction for adjusting urinary hormone concentrations. Microtiter plate competitive enzyme immunoassays were used to measure the daily values for urinary metabolites E1G and PdG as previously reported [1,2]. Urinary LH was measured in samples during the ovulatory phase of the menstrual cycle as determined by visual confirmation of a pre-ovulatory rise in E1G followed by a sustained increase in PdG. LH was determined using a coat-a-count immunoradiometric assay (Siemens Healthcare Diagnostics, Deerfield, IL). The sensitivity of the LH assay was 0.15 mIU/ml and the intra-assay and inter-assay coefficients of variation were 1.6% and 7.1%, respectively.

### Serum measurements

Blood was collected after an overnight fast before 1000 hr once during the study period. Participants were asked to lie supine for at least 15 minutes after which a blood sample was obtained via venipuncture. Samples were allowed to clot for at least 30 minutes at room temperature and then spun in a centrifuge at 4° Celsius for 15 minutes at 3000 rpm whereafter serum was transferred into appropriately labeled 1.5 mL microtubules and stored at −80° Celsius until analysis. Serum samples were assayed in duplicate for TT_3_ using an Immulite chemiluminescent assay (First Generation Immulite 1000, Siemens, Deerfield, IL). The sensitivity of the assay was 35 ng/dL and the intra-assay and inter-assay coefficients of variation were 10.3% and 13.3%, respectively. To determine FAI for screening purposes, total testosterone was measured using a radioimmunoassay kit (Siemens, Los Angeles, CA) through competitive immunoassay. The sensitivity of the assay was 0.14 nmol/L (4.0 ng/dl) and the intra-assay and inter-assay coefficients of variation were 6.4% and 7.5%, respectively. SHBG was assayed in duplicate using a chemiluminescence analyzer (First Generation Immulite 1000, Siemens, Deerfield, IL) through competitive immunoassay. The sensitivity of the assay was 0.2 nmol/L (5.76 ng/dl) and the intra-assay and inter-assay coefficients of variation were 6.4% and 8.7%, respectively.

### Statistical analysis

Using expected differences (3.2 kcal/kg body weight) and standard deviations (8.0) from De Souza et al. [2] who demonstrated significant differences in EA in exercising women with subtle menstrual cycle disturbances (ovulatory vs. luteal phase defects), a sample size of 80 participants provides adequate power (1-β = 0.80) to detect significant differences in EA among menstrual groups. All variables were tested for outliers and normality using box plot analyses and Kolmogorov-Smirnova tests of normality, respectively. Extreme outliers represented a value more than 3 times the inter-quartile range (Q3-Q1) from the upper (Q3) or lower (Q1) quartile. Data points identified as extreme outliers were removed from subsequent analyses: BMI (n = 1), LBM (n = 3), VO_2peak_ (n = 2), EEE (n = 1) and TT_3_ concentrations (n = 1). A one-way analysis of variance (ANOVA) was performed to examine differences in EA among subclinical menstrual groups (ExOv, ExIncon, ExAnov, ExOligo, ExAmen). Post hoc testing to reveal where significant differences occurred was performed using t-tests with Bonferroni correction, where P < 0.0125 was considered significant. When differences were compared between clinical menstrual groups i.e., ExEumen vs. ExAmen, an independent *t*-test was used. Chi-square analyses were performed to examine the frequency of menstrual cycle disturbances at or above, and below an EA of 30 kcal/kg LBM. Data are reported as means ± SEM, and p ≤ 0.05 was considered statistically significant. All data were analyzed using SPSS for Windows (version 18; Chicago, Ill., USA).

## Results

### Demographic, reproductive, and training characteristics of participants

Demographic, reproductive, and training characteristics for all groups of exercising women are shown in Table 1. When participants are grouped according to their clinical menstrual status (ExEumen vs. ExAmen), there were no differences in height, weight, body fat (%), fat mass, lean body mass, VO_2peak_, exercise volume, exercise frequency or exercise intensity (p > 0.05). However, significant differences in age, BMI, age of menarche and gynecological age were observed (p < 0.05). When participants’ menstrual status is characterized in more detail using hormonal measures, the groups were similar with respect to age, height, weight, BMI, body fat (%), fat mass, lean body mass, VO_2peak_, exercise volume, exercise frequency, and exercise intensity. An older age of menarche was observed in the ExAmen than in the ExOv (p = 0.036). A younger gynecological age was observed in the ExAmen than in the ExOv (p = 0.004).

**Table 1:** **Demographic, reproductive, training, and metabolic characteristics of participants categorized by exercise and menstrual status**

	**ExOv**	**ExIncon**	**ExAnov**	**ExOligo**	**ExAmen**	P-value*	**ExEumen**	P-value**
	**(n = 20)**	**(n = 13)**	**(n = 8)**	**(n = 20)**	**(n = 30)**		**(n = 41)**	
**Demographic characteristics**								
Age (years)	24.9 ± 1.1	23.6 ± 1.2	24.9 ± 2.2	22.6 ± 0.9	21.6 ± 0.6	0.068	24.5 ± 0.8	0.004
Height (cm)	164 ± 1	166 ± 2	166 ± 1	166 ± 1	166 ± 1	0.869	165 ± 1	0.589
Weight (kg)	57.8 ± 1.3	59.5 ± 1.7	60.2 ± 1.2	58.1 ± 1.4	56.9 ± 1.3	0.594	58.8 ± 0.8	0.204
BMI (kg/m^2^)	21.4 ± 0.4	22.1 ± 0.3	21.8 ± 0.5	21.0 ± 0.4	20.7 ± 0.4	0.191	21.7 ± 0.2	0.045
Body fat (%)	25.2 ± 0.8	26.4 ± 1.3	26.0 ± 1.7	25.0 ± 1.2	25.0 ± 1.0	0.905	25.7 ± 0.6	0.526
Fat mass (kg)	14.5 ± 0.6	15.7 ± 1.0	15.5 ± 1.3	14.2 ± 0.8	14.4 ± 0.7	0.743	15.1 ± 0.5	0.410
Lean body mass (kg)	41.5 ± 0.9	41.2 ± 0.6	43.5 ± 0.7	42.0 ± 1.2	40.2 ± 0.8	0.318	41.9 ± 0.5	0.076
**Reproductive characteristics**								
Age of menarche (years)	12.2 ± 0.3	12.1 ± 0.4	12.4 ± 0.5	13.4 ± 0.4	13.5 ± 0.3^a^	0.006	12.2 ± 0.2	<0.001
Gynecological age (years)	12.7 ± 1.1	11.5 ± 1.2	12.4 ± 2.3	8.8 ± 1.0	8.0 ± 0.6^a^	0.002	12.2 ± 0.8	<0.001
**Training characteristics**								
VO_2peak_ (ml/kg/min)	47.3 ± 1.1	44.5 ± 1.4	46.3 ± 2.4	45.4 ± 0.9	45.4 ± 1.6	0.841	46.2 ± 0.8	0.656
Exercise volume (min/week)	338 ± 46	360 ± 57	377 ± 63	377 ± 61	357 ± 51	0.998	353 ± 31	0.934
Exercise frequency (sessions/week)	4.2 ± 0.4	4.7 ± 0.5	5.1 ± 0.7	4.6 ± 0.4	4.4 ± 0.3	0.819	4.4 ± 0.3	0.989
Exercise intensity (kcal/min)	6.1 ± 0.5	4.9 ± 0.4	4.9 ± 1.0	5.2 ± 0.5	6.2 ± 0.4	0.149	5.5 ± 0.3	0.145
**Metabolic characteristics**								
TT_3_	92.7 ± 3.2	93.5 ± 6.4	103.0 ± 8.9	90.4 ± 5.6	79.6 ± 4.1	0.048	95.3 ± 2.9	0.002
REE	32.0 ± 0.5	30.1 ± 0.5	29.2 ± 0.8	29.2 ± 0.8^a^	29.7 ± 0.6	0.020	30.8 ± 0.4	0.112
REE/pREE	0.96 ± 0.02	0.90 ± 0.02	0.90 ± 0.03	0.87 ± 0.02^a^	0.85 ± 0.02^a^	0.005	0.92 ± 0.01	0.001

Values are the mean ± SEM.

ExOv, exercising ovulatory; ExIncon, exercising inconsistent menstrual cycles; ExAnov, exercising anovulatory; ExOligo, exercising oligomenorrhea; ExAmen, exercising amenorrhea; ExEumen, exercising eumenorrhea, includes ExOv, ExIncon and ExAnov; TT_3_, total triiodothyronine; REE, resting energy expenditure.

^a^p < 0.05 vs. ExOv.

*p-value associated with ANOVA performed to examine mean differences between ExOv, ExIncon, ExAnov, ExOligo and ExAmen groups.

**p-value associated with independent T-tests performed to examine mean differences between ExAmen and ExEumen groups.

### Menstrual status

**Figure 1:**
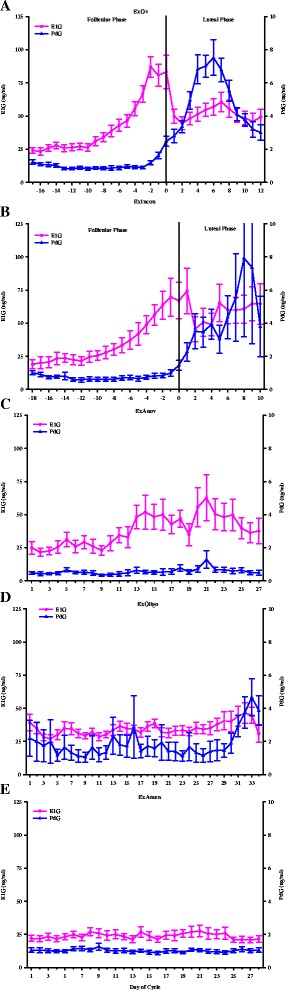
**Menstrual characteristics of participants.** This figure displays the menstrual characteristics of exercising ovulatory (ExOv) **(A)**, exercising with inconsistent menstrual cycles (ExIncon) **(B)**, exercising anovulatory (ExAnov) **(C)**, exercising oligomenorrheic (ExOligo) **(D)**, and exercising amenorrheic (ExAmen) **(E)** participants. The estrone-1-glucuronide (E1G) (ng/ml) and pregnanediol-glucuronide (PdG) (μg/ml) data for the ExOv and ExIncon participants are aligned by the day of the LH peak, defined as day 0. The number of days depicted for the ExOv, ExIncon, ExAnov, and ExOligo participants represents the mean cycle length for these participants. The number of days depicted for the ExAmen participants represents the menstrual collection period, 28 days. Data are reported as mean ± SEM of the one to three menstrual cycles per participant such that each participant’s data are represented once in the figure.

Of the total 91 exercising women in this study, 45% (41/91) were categorized as ExEumen, 22% (20/91) were categorized as ExOligo, and 33% (30/91) were categorized as ExAmen. Composite graphs depicting the urinary metabolites, E1G and PdG, of the ExOv, ExIncon, ExAnov, ExOligo and ExAmen groups are presented in Figure 1. In the ExOv (Figure 1A) group, a mid-cycle E1G peak (>35 ng/ml) during the follicular phase followed by a peak PdG concentration (>5 μg/ml) during the luteal phase was observed. In the ExIncon group (Figure 1B), a combination of a minimal increase in E1G (<35 ng/ml) during the follicular phase, inadequate PdG concentrations (<5 μg/ml) during the luteal phase, and shortened luteal phases (<10 days) was observed. In the ExAnov group (Figure 1C), a minimal increase in E1G during the follicular phase followed by inadequate PdG concentrations (<2.5 μg/ml) during the luteal phase was observed. In the ExOligo group (Figure 1D), elevated and erratic E1G concentrations were observed. Lastly, in the ExAmen group (Figure 1E), chronically suppressed E1G and PdG concentrations were observed. The average duration of amenorrhea for the ExAmen group was 252.2 ± 35.1 days.

### Energy availability and menstrual status

**Figure 2:**
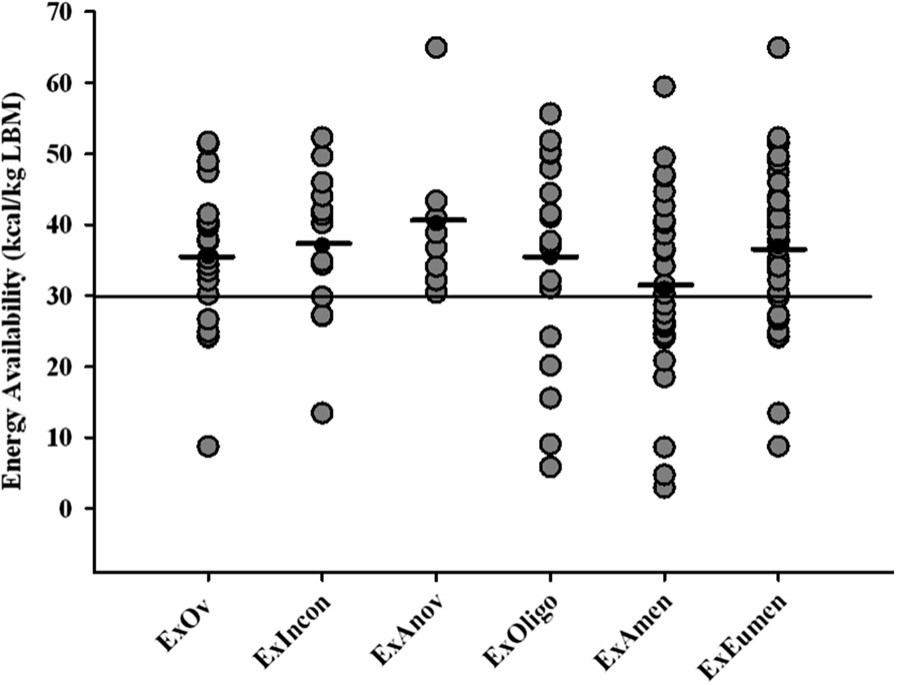
**Individual energy availability of participants.** This figure displays the individual energy availability data for each of the exercising with ovulatory menstrual cycles (ExOv), exercising with inconsistent menstrual cycles (ExIncon), exercising with anovulatory menstrual cycles (ExAnov), exercising with oligomenorrhea (ExOligo), and exercising with amenorrhea (ExAmen) participants during the study period. Black bar denotes group mean. Horizontal line across plot highlights previously established threshold for energy availability of 30 kcal/kg/LBM.

EI, EEE and EA of participants categorized by exercise and menstrual status are shown in Table 2. When participants were grouped according to their clinical menstrual status, a significantly lower EI was observed in the ExAmen vs. ExEumen groups (p = 0.013) and mean EA distinguished the groups (p = 0.036). When participants were grouped according to detailed reproductive hormone profiles, i.e., hormonal status, the ability of EA to discriminate menstrual status was poor. An EA of 30 kcal/kg LBM did not discriminate menstrual status such that the proportion of participants with at least one of the subclinical menstrual disturbances was not dependent on whether EA was above or below 30 kcal/kg LBM (*χ*^2^ = 0.557, p = 0.456). There was no difference in mean EA among the menstrual groups (F = 1.2, p = 0.297). Out of 62 of the exercising women with an EA at or above 30 kcal/kg LBM, 76% (47/62) exhibited menstrual disturbances [ExIncon (9/62), ExAnov (8/62), ExOligo (15/62) and ExAmen (15/62)] (Figure 2). Out of 29 participants with an EA below 30 kcal/kg LBM, 83% (24/29) exhibited menstrual disturbances [ExIncon (4/29), ExAnov (0/29), ExOligo (5/29) and ExAmen (15/29)] (Figure 2). When the menstrual status of participants with an EA < 30 kcal/kg LBM was compared, no differences were detected between the groups (F = 1.2, p = 0.345) and thus a relation between EA and the severity of menstrual disturbances could not be established.

**Table 2:** **Energy intake, exercise energy expenditure and energy availability of participants categorized by exercise and menstrual status**

	**ExOv**	**ExIncon**	**ExAnov**	**ExOligo**	**ExAmen**	P-value*	**ExEumen**	P-value**
	**(n = 20)**	**(n = 13)**	**(n = 8)**	**(n = 20)**	**(n = 30)**		**(n = 41)**	
Energy intake (kcal/d)	1957 ± 114	1954 ± 153	2091 ± 167	1859 ± 132	1677 ± 95	0.203	1991 ± 80	0.013
EEE (kcal/d)	470 ± 43	393 ± 59	357 ± 90	372 ± 48	413 ± 36	0.559	426 ± 32	0.810
EA (kcal/kg/LBM)	35.5 ± 2.4	37.1 ± 3.0	40.1 ± 3.9	35.4 ± 3.2	30.9 ± 2.4	0.297	36.9 ± 1.7	0.036

Values are the mean ± SEM.

ExOv, exercising ovulatory; ExIncon, exercising inconsistent menstrual cycles; ExAnov, exercising anovulatory; ExOligo, exercising oligomenorrhea; ExAmen, exercising amenorrhea; ExEumen, exercising eumenorrhea, includes ExOv, ExIncon and ExAnov; EEE, exercise energy expenditure; EA, energy availability.

*, p-value associated with ANOVA performed to examine mean differences between ExOv, ExIncon, ExAnov, ExOligo and ExAmen groups.

**, p-value associated with independent T-tests performed to examine mean differences between ExAmen and ExEumen groups.

### Other measures of metabolic status and relation to menstrual status

Metabolic characteristics for all groups of exercising women are shown in Table 1. When participants were grouped according to clinical menstrual status, lower TT_3_ concentrations (79.6 ± 4.1 vs. 95.3 ± 2.9 ng/mL, p = 0.002) and ratio of REE/pREE (0.85 ± 0.02 vs. 0.92 ± 0.01, p = 0.001) in the ExAmen group was observed compared to the ExEumen group. A lower REE was observed in the ExOligo than in the ExOv (p = 0.021). A lower REE/pREE was observed in the ExOligo (p = 0.044) and ExAmen (p = 0.002) than in the ExOv.

## Discussion

This study is the first to demonstrate that EA, defined as EI minus EEE normalized to kilograms of LBM and assessed using conventional methods, i.e., self-reported diet logs, exercise logs and heart rate monitoring, is not associated with menstrual status when the entire spectrum of menstrual disturbances is considered. However, EA does discriminate clinical extremes, i.e., amenorrhea (no menses in past 90 days) from eumenorrhea (regular menstrual cycle intervals of 26–35 days in length) in exercising women. The lower TT_3_ concentrations and ratio of REE/pREE observed in our exercising women with amenorrhea corroborate that our participants were exhibiting adaptations to chronic energy deficiency [7,30]. From a clinical perspective, this finding suggests that EA may be a meaningful measurement for exercising women to assess their energy status and consequently risk for the Female Athlete Triad such that substantial decreases in their EA values would be suggestive of a greater risk for severe clinical menstrual disturbances, a negative clinical condition of the Female Athlete Triad. Recommendations to monitor EA should however include the caveat that it requires recording of diet and exercise logs for several days or other technology to assess dietary intake and EEE kcals and knowledge of how to interpret EI, EEE and EA values. Moreover, these measurements need to be performed several times throughout the training season when athletes move in and out of training periodization. The latter issues reflect realistic challenges to incorporating EA measurements into an athlete’s regimen.

Our findings contrast those of other investigators who have examined EA [20] or its components [30-32] in exercising women. Schaal et al. [20] did not observe differences in EA between eumenorrheic and amenorrheic endurance trained athletes. Moreover, of investigators who examined the components of EA [30-32], neither Myerson et al. [30], Wilmore et al. [31], nor Laughlin and Yen [32] observed significant differences in EI, EEE, or body composition between amenorrheic and eumenorrheic athletes. Differences in the type of participants studied (e.g. recreationally active females vs. female athletes) and whether weight restrictions exist (e.g. aesthetic vs. weight vs. endurance sports) as well as smaller sample sizes of these studies [20,30-32] may account for the lack of observed differences in EA in association with menstrual status. In this study, we observed differences in EA between exercising women with eumenorrhea versus exercising women with amenorrhea when a larger sample of exercising women were included in the analyses (n = 71). Inconsistencies in the calculation of EA and the variability in methods used to estimate the components of EA and or the characterization of menstrual status may also contribute to conflicting findings.

The current study is the first to test the concept that an EA threshold of 30 kcal/kg LBM discriminates menstrual status when EA is assessed with self-reported diet logs, exercise logs and heart rate monitoring in a large group of exercising women studied under free living conditions. Additionally, this study is the first to examine whether menstrual cycle disturbances progress in severity from luteal phase defects to anovulatory menstrual cycles to oligomenorrhea, and amenorrhea as EA becomes incrementally lower below an EA of 30 kcal/kg LBM. We did not observe a threshold of 30 kcal/kg LBM below which EAMD occurred or any association between severity of menstrual disturbances and EA. Indeed, participants with an EA below 30 kcal/kg LBM exhibited the entire spectrum of menstrual disturbances i.e., inconsistent combinations of ovulatory, luteal phase defects, and anovulatory menstrual cycle (14%) as well as oligomenorrhea (17%) and amenorrhea (52%). The observation that all types of EAMD were observed below 30 kcal/kg/LBM may indicate that there is not a clear association between the magnitude of disruption in LH pulsatility [14] and menstrual disturbances, or that the association between a threshold of EA and menstrual disturbances might manifest over longer time periods of exposure to given levels of EA. Since our study captured only a relatively short sampling period (up to three menstrual cycles or monitoring period), it is possible that a longer time period of observation would have revealed a stronger association between EA and specific types of menstrual disturbances.

In a short term study manipulating EI and EEE which led to significant disruptions in LH pulsatility below an EA of 30 kcal/kg LBM, Loucks et al. [14] used a clinical dietary product (Ensure, Ross Laboratories, Columbus, OH) to control EI and indirect calorimetry to assess EEE. Conversely in the current study, we assessed EI and EEE using more conventional methods for exercising women. Namely, self-reported diet logs, exercise logs, and heart rate monitoring were used to assess EA. Several studies have shown inaccuracies when measuring EI with self-reported diet logs, particularly related to under reporting [33] and EEE with self-reported exercise logs [33]. Heart rate monitoring has been shown to estimate total EEE within −4.0% to 11.4% in females when compared to doubly labeled water which is the gold standard for measuring energy expenditure in free living conditions. Although heart rate monitoring provides an objective assessment of EEE, participants must be compliant and wear the monitors for the duration of all their exercise training sessions. It is therefore possible that the inaccuracies associated with self-reported diet logs, exercise logs, and heart rate monitoring in assessing EI and EEE may have contributed to the lack of observed differences in EA among our exercising women with varying menstrual status (ovulatory vs. EAMD). Alternatively, factors such as psychological stress [34] or hyperandrogenemia [25] may also account for a proportion of the observed EAMD and this could thus weaken any suspected association between EA and menstrual disturbances. However, differences in EA were observed among participants with eumenorrheic and amenorrheic menstrual cycles suggesting that the conventional methods used in the current study to assess EA are able to discriminate more clinically extreme categories of menstrual status.

We observed lower EA values on average in our exercising women in comparison to those reported in the literature for sedentary women [2]. These lower values may be attributable to under eating relative to energy expenditure. Findings from studies examining the coupling between food intake and EEE [35-37] suggest that exercise may be linked with inadvertent under eating and low EI. The lack of an increase in food intake in response to an acute exercise-induced energy expenditure i.e. "inadequate compensation" has been observed in obese and non-obese men and women [35-37]. The above studies [35-37] suggest that inadvertent dietary compensation to match EEE may have contributed to the lower overall mean EA (35 kcal/kg LBM) observed in our exercising women when compared to the EA values we calculated from the reported EI, EEE, and body composition of the sedentary women of Myerson et al. [30] (39 kcal/kg FFM) and Laughlin and Yen [32] (38 kcal/kg LBM). Regarding the relation between energy intake and exercise in our own data, we found that as EEE increased, EA decreased (r = −0.245, p = 0.020). This may suggest that inadequate dietary compensation increases as the volume of exercise increases.

Although our exercising women exhibited a lower overall mean EA than sedentary women [30,32], our mean EA values (31–40 kcal/kg LBM)) are higher than other studies of exercising women. For example, using EI, training mileage, and fat free mass from several studies of female amenorrheic runners, Loucks et al. [38] estimated EA values ranging from 12 to 29 kcal/kg FFM in female athletes. Schaal et al. [20] and Reed et al. [21] observed EA values below 30 kcal/kg LBM in exercising women with amenorrhea, albeit in a much smaller sample of athletes than our investigation. Lastly, using reported EI, EEE, and FFM, we estimated an EA value of 28 kcal/kg FFM for the amenorrheic runners of Myerson et al. [30]. Since we observed a negative relationship between EA and EEE, suggestive of the presence of an uncoupling mechanism between EI and EEE [35-37], it is possible that inadequate EI compensation to match lower EEE resulted in higher EA values (>30 kcal/kg LBM) when compared to other studies where the training volume of the participants and thus EEE was higher [20] than in our exercising women. Using this rationale, the higher mean EEE of Schaal et al. [20] (1300 kcal/d) and Myerson et al. [30] (537 kcal/d) would explain the lower EA these investigators reported [20,30]. These investigators [20,30] also observed negative eating attitudes including high body shape concerns and total eating disorder scores in their female athletes. Aberrant eating attitudes have been shown to predispose women to consume less EI [5]. It is therefore also possible that higher EEE in combination with negative eating attitudes might have contributed to the lower EA values documented in these amenorrheic exercising women [20,30].

Limitations of this study include the fact that EI and some EEE was collected using self-report 3-day diet and 7-day exercise logs. Several studies have shown inaccuracies when measuring EI using self-reported food records, particularly related to underreporting [33]. Further, 3-day diet logs may not provide the best indication of typical EI in exercising women as their dietary intakes may change with their weekly training routine. We did not eliminate possible under reporters in the current study as: a gold standard for accounting for under reporters does not exist; other prior publications examining EA in exercising females have not employed a cut off method to eliminate under reporters from analyses [4,20,39]; a cut off for under reporting does not identify over reporters; and, under reporting can also be a result of intentional caloric restriction. However, all of the participants were trained by on-site registered dietitians on how to accurately record food intake. The EA values reported in this study were normalized to kilograms of LBM. EA values normalized to kilograms of LBM may be slightly greater than if normalized to kilograms of FFM. The units of reference should be considered when comparing findings regarding the association between EA and menstrual status. An important feature of this study is that the data analyzed likely represent the largest dataset available in exercising women to date where menstrual disturbances have been confirmed with measurements of estrogen, progesterone, and luteinizing hormone metabolites from daily urine samples.

## Conclusion

This study was the first to show that EA, defined as EI minus EEE normalized to kilogram of LBM, did not discriminate subclinical menstrual status in a large sample of trained, exercising women with varying menstrual cycles, but did discriminate amenorrhea from menstrual cycles with regular bleeding intervals when using conventional methods i.e., self-reported diet logs, exercise logs, and heart rate monitoring to asses EA. Lower TT_3_ concentrations and ratio of REE/pREE were observed in our exercising amenorrheic women indicating that our participants likely were exhibiting adaptations to chronic energy deficiency. We propose that substantial decreases in EA calculated using the above methods may be a useful index for exercising women to monitor their risk for amenorrhea, but not less severe or subtle menstrual disturbances. Future studies should focus on identifying ways to improve field measures of EI and EEE. More precise techniques to measure these components over longer time periods may improve the ability of EA in discriminating subclinical menstrual disturbances and those at risk for the Female Athlete Triad.

## Abbreviations

BMI: Body mass index
EA: Energy availability
EEE: Exercise energy expenditure
E1G: Estrone-1-glucoronide
ExAmen: Exercising amenorrhea
ExAnov: Exercising anovulatory
ExEumen: Exercising eumenorrhea
ExIncon: Exercising inconsistent menstrual cycles
ExOligo: Exercising oligomenorrhea
ExOv: Exercising ovulatory
EI: Dietary energy intake
FFM: Fat free mass
LBM: Lean body mass
LH: Luteinizing hormone
MET: Metabolic equivalents
PdG: Pregnanediol glucuronide
REE: Resting energy expenditure
TT_3_: Total triiodothyronine
